# Design of an Adaptive Fixed-Time Fast Terminal Sliding Mode Controller for Multi-Link Robots Actuated by Pneumatic Artificial Muscles

**DOI:** 10.3390/biomimetics10010037

**Published:** 2025-01-08

**Authors:** Hesam Khajehsaeid, Ali Soltani, Vahid Azimirad

**Affiliations:** 1 Warwick Manufacturing Group, The University of Warwick, Coventry CV4 7EQ, UK; 2 School of Electronics and Computer Science, University of Southampton, Southampton SO17 1BJ, UK; ali.soltani@soton.ac.uk; 3 School of Engineering, University of Kent, Canterbury CT2 7NZ, UK; v.azimirad@kent.ac.uk

**Keywords:** pneumatic artificial muscle, continuum mechanics, sliding mode control, fixed time, fast terminal

## Abstract

Pneumatic artificial muscles (PAMs) are flexible actuators that can be contracted or expanded by applying air pressure. They are used in robotics, prosthetics, and other applications requiring flexible and compliant actuation. PAMs are basically designed to mimic the function of biological muscles, providing a high force-to-weight ratio and smooth, lifelike movement. Inflation and deflation of these muscles can be controlled rapidly, allowing for fast actuation. In this work, a continuum mechanics-based model is developed to predict the output parameters of PAMs, like actuation force. Comparison of the model results with experimental data shows that the model efficiently predicts the mechanical behaviour of PAMs. Using the actuation force–air pressure–contraction relation provided by the proposed mechanical model, a dynamic model is derived for a multi-link PAM-actuated robot manipulator. An adaptive fixed-time fast terminal sliding mode control is proposed to track the desired joint position trajectories despite the model uncertainties and external disturbances with unknown magnitude bounds. Furthermore, the performance of the proposed controller is compared with an adaptive backstepping fast terminal sliding mode controller through numerical simulations. The simulations show faster convergence and more precise tracking for the proposed controller.

## 1. Introduction

Pneumatic artificial muscles (PAMs) are flexible actuators that can expand/contract when air pressure is applied. They are used in robotics, prosthetics, and other applications requiring lightweight and compliant actuation. PAMs typically consist of a rubber bladder surrounded by a braided mesh or fabric. When air is introduced into the bladder, the muscle expands radially and contracts in length. This contraction generates pulling force, similar to how biological muscles work. The pulling force and length change depend on the applied air pressure as well as the properties of the bladder and mesh. PAMs are soft and flexible, allowing them to conform to various shapes and work safely in close proximity to humans. The inflation and deflation of the muscle can be controlled rapidly, allowing for fast actuation.

PAMs were originally invented by Joseph L. McKibben in the 1950s to create an actuator for prosthetic devices. Later, the idea was further developed and refined by various researchers. In the 1980s, interest in soft robotics and biomimetic actuators grew, leading to more research and development in pneumatic muscles. Nowadays, PAMs are used in a wide range of applications. They are used in soft robotics to create robots that are safe for human interaction, adaptable, and capable of complex movements. In humanoid robots, PAMs can be used to replicate human-like motions in the arms, legs, or facial muscles, allowing for more realistic expressions and movements [1,2]. PAMs are used in prosthetic devices to create more natural and responsive movements, mimicking the action of human muscles. In wearable exoskeletons, PAMs provide assistance to people with mobility impairments. PAMs can be incorporated into rehabilitation devices to provide variable resistance or assist in movement training for physical therapy [3,4].

There are also anisotropic PAMs whose mechanical properties depend on the direction of loading/actuation. Anisotropic PAMs are designed to better suit specific applications; for example, they may be stiffer along one direction to resist loads while remaining more flexible in others to allow controlled motion [5,6]. These muscles often incorporate advanced materials or asymmetric geometries in their design, such as non-uniform braiding angles, tailored fiber orientations, or segmented bladder materials. These actuators can be designed to match the biomechanical properties of a target system, such as mimicking human muscle anisotropy in robotic prosthetics or exoskeletons [7,8]; however, their mechanical modelling is more complicated and will also need extra testing to calibrate material models. For example, directional tensile tests will be needed to measure the directional stiffness as well as lateral contractions in different directions. They also require advanced control algorithms to account for directionally varying dynamics. Anisotropic PAMs can perform tasks like bending, twisting, or asymmetric contraction, which are difficult to achieve with isotropic designs.

PAMs exhibit highly nonlinear behaviour due to the nonlinear mechanical properties of the rubber bladder, the complicated behaviour of the outer shell, and the compressibility of air. To employ PAMs in engineering applications, it is necessary to effectively understand the nonlinear elastic behaviour of the bladder first. Early attempts to model the behaviour of PAMs focused on developing analytical models to describe the relationship between input air pressure, muscle contraction, and generated force. The most well-known model is the Geometric Model developed in the 1950s by Joseph L. McKibben, which laid the foundation for understanding the basic mechanical properties of PAMs. To improve modelling accuracy, some researchers have proposed (semi-) empirical models that incorporate experimental data. Tondu and Lopez introduced a semi-empirical model that combines geometric assumptions with experimental force–displacement data [9]. Their model considers the nonlinear elasticity of the bladder material and the effect of the braided sleeve. Reynolds et al. proposed a phenomenological model that fits experimental force–strain data of PAMs [10]. While this approach offers high accuracy within the range of data used for fitting, it lacks generalisability outside the tested conditions. Doumit and Leclair developed a stiffness model to relate the air pressure, geometry of muscle, and friction in the muscle wall [11]. Soleymani and Khajehsaeid proposed a mechanical model for PAMs that predicts the stiffness, output force, and contraction of the muscle during the actuation course [12].

De Volder et al. used the Finite Element Method (FEM) to model the behaviour of PAMs [13]. Their model provides insights into the stress and strain distributions within the muscle and helps optimise the muscle design for specific applications. FEM-based models offer high accuracy but are computationally demanding and often require some simplifications for practical use. Recent research has focused on developing more advanced nonlinear and hybrid models that combine different modeling approaches to capture the full range of PAMs’ behaviour. Polygerinos et al. proposed a hybrid model combining analytical and numerical techniques to account for both the material nonlinearities and geometric complexities of PAMs [14]. Their model integrates FEM with analytical equations derived from the muscle geometry, providing a balance between accuracy and computational efficiency. Nguyen et al. developed a nonlinear viscoelastic model to describe the time-dependent behaviour of PAMs [15]. Their model is particularly useful for applications where PAMs are subject to prolonged loading or cyclic operations.

Dynamic models are crucial for understanding the behaviour of PAMs in time-dependent scenarios, such as in robotic or prosthetic applications where rapid movement and force adjustments are needed. Davis and Caldwell developed a dynamic model for PAMs that considers both the inertial effects of the muscle and the air flow dynamics through the pneumatic system [16]. Due to their high power-to-noise ratio and flexibility, PAMs have been widely used as the actuators of robotic systems in bio-robotics [17,18] and medical [19,20,21] and industrial applications [22]. Kawamura et al. designed a humanoid robot with two six-DOF arms where multiple PAMs actuate the joints antagonistically [17]. Haghshenas-Jaryani addressed the dynamic modeling and feedback linearisation control of the planar locomotion of an artificial-muscle-driven snake-like robot [18]. Dragone et al. presented a bio-robotic joint with controllable compliance by implementing PAMs for actuation [19]. Chi et al. developed an antagonistic PAM-driven rehabilitation device [20]. Another study considered the feasibility of the application of a single PAM to actuate a 1-DOF lower-limb therapy robotic system [21]. Lin et al. designed an in-pipe inspection robot actuated by a single McKibben muscle actuator, demonstrating outstanding crawling performance [22].

Due to the nonlinear and complex dynamics of PAMs, controlling them is challenging, particularly when they are implemented antagonistically as the actuators of robotic manipulators. The control system should also be able to track the desired trajectories in the presence of model uncertainties and disturbances which are so likely for PAM-actuated robots. To address this problem, an adaptive control law was designed by Karnjanaparichat et al. for PAM-actuated multi-link robots. The effectiveness of the developed control law in tracking the desired position of the joints has been verified via simulations [23]. However, the pneumatic muscle was modeled as a spring, a damper, and a contractile force, which is very simplistic. Xing et al. developed a nonlinear disturbance observer-based sliding mode controller (SMC) to improve the trajectory tracking performance of a PAM-driven hand rehabilitation device [24]. In this study, the PAM characteristics were approximated by a piecewise function instead of using a mechanical model for controller design. Therefore, the proposed controller can be applied to applications requiring low pressures. Wu et al. designed a dynamic surface control (DSC) based on a nonlinear disturbance observer to improve the tracking performance of a PAM system. They evaluated the effectiveness of the proposed control method via experimental tests [25]. However, the PAM was modeled in the same way as [23], which can significantly restrict achievable performance by the proposed controller. Thanh et al. [26] designed a nonlinear PID controller by augmenting a classical PID controller with neural networks to improve the tracking performance of a two-link robot manipulator actuated by PAMs in the same configuration as [25]. Although the proposed control scheme showed satisfactory performance in the experiments, the stability of the system has been neither proven nor analysed. Ganguly et al. extended a conventional PID controller incorporating an inner pressure regulation loop for trajectory tracking of a PAM-driven SDOF robot manipulator [27]. Although in this study the pressure regulation loop is included in the control design, the performance of the controller were experimentally evaluated in tracking small angle low-speed trajectories where the nonlinearities are not significant. In [28], three control strategies, SMC, adaptive SMC, and adaptive neural network (ANN) control, were developed and compared in precise trajectory tracking of a PAM-actuated SDOF manipulator. The results suggest that the ANN is preferable in most scenarios since it does not require a complete model of the pneumatic system and is robust against the variations in PAM actuator characteristics due to fatigue or replacement. This study approximates quasi-static manipulator torque by a linear function of pressure combined with third-order polynomial functions of the angle. This can potentially degrade the level of practically achievable tracking accuracy. Qin et al. [29] proposed a robust control strategy with disturbance compensation for the hysteresis compensation and trajectory tracking of PAMs. They implemented a modified Prandtl–Ishlinskii model as a feedforward compensator for hysteresis and applied adaptive set-membership filtering (ASMF) to estimate the nonlinear terms and external disturbances. The results of the experimental tests verify the effectiveness of the proposed control scheme. However, as a linearised model of PAM is used to design the control law, the proposed controller can only guarantee local stability and would only have acceptable performance in tracking slow low-amplitude trajectories. Khajehsaeid et al. [30] extended a continuum-based mechanical model for modelling the actuation force of PAMs. They designed an adaptive backstepping fast terminal sliding mode control for high-accuracy trajectory tracking of PAM-actuated joints of multi-link planar robot manipulators in the presence of model uncertainties and external disturbances. Zhao et al. [31] designed a prescribed performance sliding mode control method to perform joint trajectory tracking for an elbow exoskeleton actuated by two antagonistic PAMs. The calculated control torque is mapped to the internal pressure of the muscle by interpolating the data collected through some static experiments, which is not efficient enough as the controller exhibits high-frequency oscillations. Duong et al. [32] proposed a discrete-time sliding mode control augmented with an adaptive fuzzy algorithm to address the unknown disturbances of a PAM-driven actuator. Although the experimental results verify the usefulness of the developed control strategy to some extent, the tracking is not very accurate because the implemented model for the PAM is not sufficiently realistic, particularly for large deformations of muscle.

In the present work, a continuum mechanics-based model is developed for isotropic PAMs. The model provides an explicit relation between contraction ratio, air pressure, and the actuation force as a set of input/output parameters for dynamic modelling and/or control purposes of PAM-actuated systems. Moreover, a dynamic model is developed for multi-link PAM-actuated robot manipulators. The dynamic model uses the proposed mechanical model to determine the required air pressure for any given load and motion input. To control the manipulator, an adaptive fixed-time fast terminal sliding mode controller is proposed to track the desired joint position trajectories despite the model uncertainties as well as external disturbances with unknown magnitude bounds. Performance of the controller is compared via numerical simulations with an adaptive backstepping fast terminal sliding mode controller proposed by the authors in another work.

## 2. Pneumatic Artificial Muscles

As discussed in Section 1, it is essential to have a reliable mechanical model for PAMs before modelling or designing any PAM-actuated device. Therefore, the first step would be choosing an existent model or developing one which fits the purpose of the modelling/design. As there is a need for an explicit force–pressure–contraction relation in this work to employ that in the controller design, the authors will try to propose a closed-form, simple but still reliable relation for this purpose. Such a model needs to satisfy the mechanical equilibrium equations and the boundary conditions imposed by the design.

### 2.1. Mechanical Modelling of PAMs in a Large Deformation Regime

**Figure 1:**
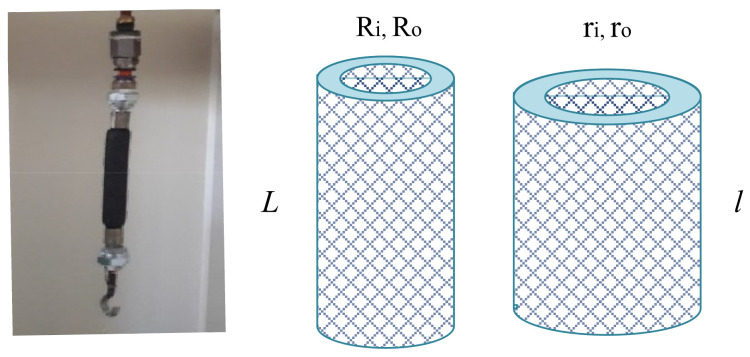
(**Left**) The designed PAM and (**Right**) the rest and deformed states.

Figure 1 shows a PAM where the inner elastomeric bladder is assumed as a thick-walled cylinder. The continuum mechanical representation of the body in the undeformed state is

(1) $$R_{i}\leq R\leq R_{o}\;\;\;\;\;,\;\;\;\;\;0\leq \Theta \leq 2\pi \;\;\;\;\;,\;\;\;\;\;0\leq Z\leq L$$

where (R,Θ,Z) are the cylindrical coordinates in the initial configuration. Upon inflation, the bladder is described by

(2) $$r_{i}\leq r\leq r_{o}\;\;\;\;\;,\;\;\;\;\;0\leq \theta \leq 2\pi \;\;\;\;\;,\;\;\;\;\;0\leq z\leq l$$

where $\left(r,\theta ,z\right)$ are the cylindrical coordinates in the deformed configuration.

Assuming that the muscle is axisymmetric, its deformation can be described by $z=\lambda_{z}Z\;\mathrm{and}\;\theta =\Theta$, where $\lambda_{z}=\frac{l}{L}$ is the axial stretch of the bladder. The mechanical behaviour of elastomers can often be described by means of hyperelastic material models. Such models provide a mathematical interpretation of the strain energy potential function (W) stored in the material due to deformation, which can be used to derive a relation for stress and strain/stretch. Due to the objectivity considerations, strain energy functions (SEFs) are defined in terms of the deformation gradient tensor F or invariants of the left or right Cauchy–Green deformation tensor B  or C.

The deformation gradient F for the bladder in the cylindrical coordinates is given by

(3) $$F=\left[\begin{array}{ccc} \frac{dr}{dR} & 0 & 0\\ 0 & \frac{r}{R}\frac{d\theta }{d\Theta } & 0\\ 0 & 0 & \frac{dz}{dZ} \end{array}\right]=\left[\begin{array}{ccc} \frac{dr}{dR} & 0 & 0\\ 0 & \frac{r}{R} & 0\\ 0 & 0 & \lambda_{z} \end{array}\right]$$

Noting $B\;={\mathrm{FF}}^{T}$, the corresponding left Cauchy–Green deformation tensor is obtained as

(4) $$B=diag\left({\left(\frac{dr}{dR}\right)}^{2},\;\;{\left(\frac{r}{R}\right)}^{2},\;{\lambda_{z}}^{2}\right)$$

Invariants of the left Cauchy–Green deformation tensor are

(5) $$\begin{array}{l} I_{1}=tr(B)=\;\;{\left(\frac{dr}{dR}\right)}^{2}+\;\;{\left(\frac{r}{R}\right)}^{2}+\;\;{\lambda_{z}}^{2}\\ I_{2}=\frac{1}{2}[(tr{\left(B\right)}^{2}-tr(B^{2})]=\;{\left(\frac{dr}{dR}\right)}^{2}\cdot \;{\left(\frac{r}{R}\right)}^{2}+\;{\left(\frac{dr}{dR}\right)}^{2}\cdot \;\;{\lambda_{z}}^{2}+{\left(\frac{r}{R}\right)}^{2}\cdot \;{\lambda_{z}}^{2}\\ I_{3}=\det(B)=\;{\left(\frac{dr}{dR}\cdot \;\frac{r}{R}\cdot \;\lambda_{z}\right)}^{2} \end{array}$$

It is often assumed that $I_{3}=1$ because elastomers are nearly incompressible materials [33]. Integrating the third equation of (5), we conclude that $R^{2}=\lambda_{z}\;(r^{2}-\Phi )$ where Φ is the integration constant which can be determined using the boundary conditions of the problem. Also, we can re-write the invariant as:

(6) $$I_{1}=Rr^{-1}{\lambda_{z}}^{-1}+r^{2}R^{-2}+{\lambda_{z}}^{2},\;\;\;\;\;I_{2}={\lambda_{z}}^{-2}+r^{-2}R^{2}+r^{2}R^{-2}{\lambda_{z}}^{2},\;\;\;\;\;\;\;I_{3}=1$$

The incompressibility condition implies that ${r_{i}}^{2}=\;{r_{o}}^{2}-{\lambda_{z}}^{-1}({R_{o}}^{2}-{r_{i}}^{2})$.

For an incompressible isotropic material, the Cauchy stress can be written as [34]

(7) $$\sigma =-P_{h}+2W_{1}B+2W_{2}\;(I_{1}B-B^{2})\;$$

where $W_{1}$ and $W_{2}$ are derivatives of W with respect to $I_{1}$ and $I_{2}$, respectively, and $P_{h}$ is the hydrostatic pressure which should be determined considering the boundary conditions. Thus, the Cauchy stress is given by

(8) $$\begin{array}{l} \sigma =-P_{h}I+2W_{1}diag\left({\left(\frac{R}{r\lambda_{z}}\right)}^{2},\;\;{\left(\frac{r}{R}\right)}^{2},\;{\lambda_{z}}^{2}\right)+\\ 2W_{2}\left(\left({\left(\frac{R}{r\lambda_{z}}\right)}^{2}+\;\;{\left(\frac{r}{R}\right)}^{2}+\;{\lambda_{z}}^{2}\right)\cdot diag\left({\left(\frac{R}{r\lambda_{z}}\right)}^{2},\;\;{\left(\frac{r}{R}\right)}^{2},\;{\lambda_{z}}^{2}\right)-diag\left({\left(\frac{R}{r\lambda_{z}}\right)}^{4},\;\;{\left(\frac{r}{R}\right)}^{4},\;{\lambda_{z}}^{4}\right)\right) \end{array}$$

### 2.2. Boundary Conditions

**Figure 2:**
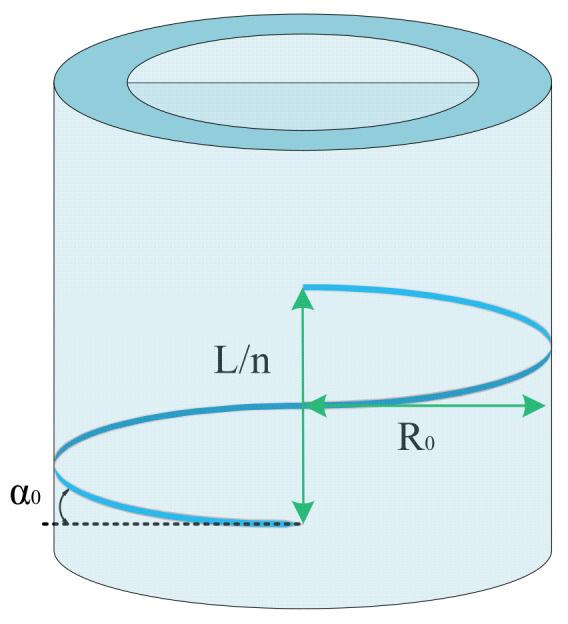
Schematic representation of a PAM and a single thread.

As shown in Figure 2, due to the relatively high stiffness of the braid material, the length of threads remains almost unaltered through the actuation course. To determine the outer radius after inflation, one can use the following geometric relation:

(9) $$L^{2}+{\left(2\pi R_{o}n\right)}^{2}=l^{2}+{\left(2\pi r_{o}n\right)}^{2}$$

In (9), n is the number of turns of a single thread around the PAM axis, which is calculated using the angle between the braid and the radial direction at rest $\left(\alpha_{0}\right)$ or deformed states (α):

(10) $$\;n=\frac{L}{2\pi R_{o}\tan\alpha_{0}}=\frac{l}{2\pi r_{o}\tan\alpha }$$

Substituting (10) into (9), the outer radius after inflation is calculated:

(11) $$r_{o}=\sqrt{\frac{L^{2}(1-{\lambda_{z}}^{2})+4\pi^{2}n^{2}{R_{o}}^{2}}{4\pi^{2}n^{2}}}$$

Now, the static equilibrium equations can be considered [35]:

(12) $$\frac{d\sigma_{r}}{dr}-\frac{1}{r}(\sigma_{\theta }-\sigma_{r})=0\;\;\Rightarrow \;\;\int_{r_{i}}^{r}d\sigma_{r}=\;\sigma_{r}(\;r\;)-\sigma_{r}(\;r_{i}\;)=\int_{r_{i}}^{r}\frac{\sigma_{\theta }-\sigma_{r}}{r}\;dr$$

The radial stress at the inner surface is $\sigma_{r}(r_{i})=-P_{air}$. To solve the equilibrium equation, one should describe the stress components in terms of an appropriate strain energy function [36]. At this stage, any standard SEF can be used; however, it is important to note the strain/stretch range in the particular application under study. For the PAMs used in robotic and rehabilitation applications, the strain range is often under 50% which implies that there is no need for complicated higher-order SEFs. Even though they might provide slightly better accuracy in results, the complication imposed into the formulation and the numerical implementation of the model is much more important. For the given range of contractions, one can confidently use the Neo-Hookean or Mooney–Rivlin SEFs, which facilitate solving the equilibrium equations. Here, the Neo-Hookean function is chosen due to its mathematical simplicity:

(13) $$W=\frac{\mu }{2}(I_{1}-3)$$

where μ is the material shear modulus. Therefore, resulting stresses take the following forms:

(14) $$\sigma_{r}=\mu \;\frac{R^{2}}{{\lambda_{z}}^{2}r^{2}}-P_{h}\;\;,\;\;\;\;\sigma_{\theta }=\mu \frac{r^{2}}{R^{2}}-P_{h}\;\;,\;\;\;\;\;\sigma_{z}=\mu {\lambda_{z}}^{2}-P_{h}$$

Using (14), Equation (12) can be written as

(15) $$\sigma_{r}=-P_{air}+\int_{r_{i}}^{r}\;\frac{\sigma_{\theta }-\sigma_{r}}{r}\;dr\;=-P_{air}+\frac{\mu }{2\lambda_{z}}\ln\frac{r^{2}-\Phi }{{r_{i}}^{2}-\Phi }-\frac{\mu }{\lambda_{z}}\ln\frac{r}{r_{i}}+\frac{\mu }{2\lambda_{z}}\Phi \left(\frac{1}{{r_{i}}^{2}}-\frac{1}{r^{2}}\right)$$

Omitting $P_{h}$, (14) and (15), the stress components can be determined as

(16) $$\sigma_{\theta }=-P_{air}+\frac{\mu }{2\lambda_{z}}\left(\ln\frac{r^{2}-\Phi }{{r_{i}}^{2}-\Phi }-2\ln\frac{r}{r_{i}}+\Phi \left(\frac{1}{{r_{i}}^{2}}-\frac{1}{r^{2}}\right)+\frac{2\Phi (2r^{2}-\Phi )}{r^{2}(r^{2}-\Phi )}\right)$$

(17) $$\sigma_{z}=-P_{air}+\frac{\mu }{2\lambda_{z}}\left(\ln\frac{r^{2}-\Phi }{{r_{i}}^{2}-\Phi }-2\ln\frac{r}{r_{i}}+\Phi \left(\frac{1}{{r_{i}}^{2}}-\frac{1}{r^{2}}\right)+2{\lambda_{z}}^{3}-2+\frac{2\Phi }{r^{2}}\right)$$

Integrating (16) and (17), the resultant circumferential and axial forces are given:

(18) $$F_{\theta }=l\int_{r_{i}}^{r_{o}}\sigma_{\theta }dr=l\;\left[-P_{air}(r_{o}-r_{i})+\frac{\mu }{2\lambda_{z}}r_{o}\ln\Psi -\frac{\mu }{\lambda_{z}}r_{o}\ln\frac{r_{o}}{r_{i}}+\frac{\mu }{2\lambda_{z}}\Phi \left(\frac{r_{o}}{{r_{i}}^{2}}+\frac{1}{r_{o}}-\frac{2}{r_{i}}\right)\right]$$

(19) $$\begin{array}{l} F_{z}=\int_{0}^{2\pi }\int_{r_{i}}^{r_{o}}\sigma_{z}rdrd\theta =-\Phi \frac{\mu }{\lambda_{z}}\pi \ln\frac{r_{o}}{r_{i}}-\mu \Phi \pi \left(\frac{1}{2\lambda_{z}}-\lambda_{z}\right)\ln\Psi +\pi \left(\mu \lambda_{z}-P_{air}\right)\left({r_{o}}^{2}-{r_{i}}^{2}\right)\\ +2\Phi \frac{\mu }{\lambda_{z}}\pi \left(\frac{{r_{o}}^{2}}{{r_{i}}^{2}}-1\right)+{r_{i}}^{2}\frac{\pi \mu }{{\lambda_{z}}^{2}}\left(1-{r_{o}}^{2}\right)\;\;\; \end{array}$$

where $\Psi =\frac{{r_{o}}^{2}-\Phi }{{r_{i}}^{2}-\Phi }$. The actuation force can be calculated by means of (19) as a control parameter.

### 2.3. The Actuation Force

**Figure 3:**
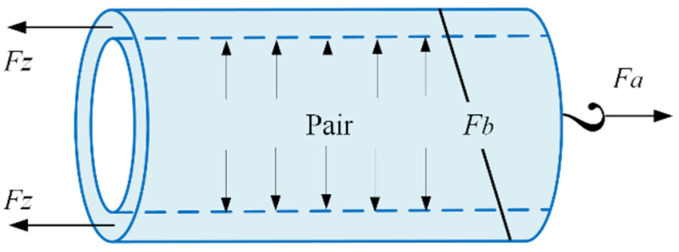
Free-body diagram of a pneumatic artificial muscle.

The equilibrium equations in the circumferential and axial directions of the PAM, respectively, result in the following equations (see Figure 3):

(20) $$\begin{array}{l} F_{\theta }+nF_{b}\cos\alpha_{2}=P_{air}r_{i}l\\ F_{z}+F_{b}\sin\alpha_{2}-F_{a}=P_{air}\pi {r_{i}}^{2} \end{array}$$

where $F_{b}$ is the tension force in the braid and $F_{a}$ is the PAM actuation force. By solving (20) for $F_{a}$, we have:

(21) $$F_{a}=F_{z}-P_{air}\pi r_{i}^{2}+\left(\frac{P_{air}r_{i}l-F_{\theta }}{n}\right)\tan(\alpha )$$

Recalling (10) and utilising (21), one can calculate the pressure ($P_{air}$) required in the controller design:

(22) $$P_{air}=\frac{2\pi n^{2}r_{o}\left(F_{a}-F_{z}\right)+F_{\theta }\lambda_{z}L}{r_{i}{\lambda_{z}}^{2}L^{2}-2\pi^{2}r_{i}^{2}n^{2}r_{o}}$$

Substituting (18) and (19) into (22), we have:

(23) $$P_{air}=\frac{\mu }{\lambda_{z}\left({r_{o}}^{2}-\frac{L^{2}{\lambda_{z}}^{2}}{2\pi^{2}n^{2}}\right)}\left(\begin{array}{l} -2\Phi \ln\frac{r_{o}}{r_{i}}-\Phi (1-2{\lambda_{z}}^{2})\ln\Psi -2{r_{o}}^{2}\ln\frac{r_{o}}{r_{i}}-\\ \frac{\left({r_{o}}^{2}-{r_{i}}^{2})(2{r_{i}}^{2}-\Phi \lambda_{z}-2a^{2}{\lambda_{z}}^{3}\right)}{{r_{i}}^{2}\lambda_{z}}-\frac{\lambda_{z}}{\pi \mu }\left(\frac{1}{2\beta }-F_{a}\right) \end{array}\right)$$

where $\beta =\mu n^{2}L{r_{o}}^{2}\left(\ln\Psi \frac{{r_{i}}^{2}}{{r_{o}}^{2}}+\Phi \left(\frac{{r_{i}}^{2}-{r_{o}}^{2}}{{r_{i}}^{2}{r_{o}}^{2}}\right)\right)$

Equation (23) can be used to determine the required internal pressure $P_{air}$ as the control signal.

## 3. Dynamic Model of PAM-Actuated Robotic Manipulators

This section derives a mathematical model for robotic manipulators with joints actuated by pairs of antagonistic PAMs. First, the actuation torques of the joints are calculated in terms of the internal pressure of the muscles; then, the initial settings of the PAMs and the associated internal pressure regulation strategy are introduced. Finally, the general model for multi-link manipulator dynamics is represented according to the adopted strategy to derive the dynamic model of the system.

### 3.1. Antagonistic Joint Actuation by PAMs

**Figure 4:**
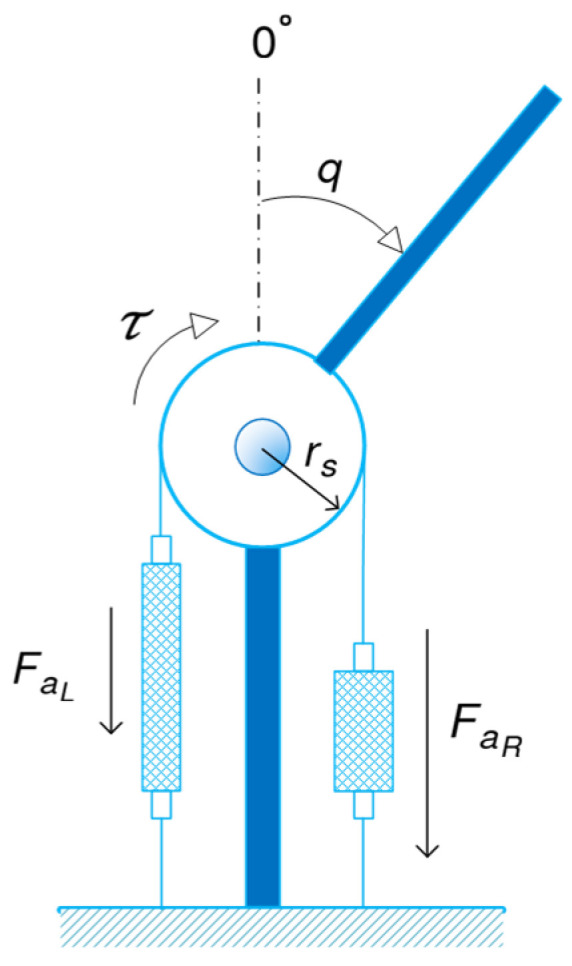
Antagonistic joint actuation by a pair of PAMs.

Figure 4 shows the antagonistic configuration used to actuate the joints of the manipulators. The physiological model of bicep–tricep systems inspires the configuration. To maximise the workspace, the initial contractions of the right and left muscles are set as $\Delta L_{R0}=\Delta L_{L0}=\pi r_{s}$ so that they are in the full-extension at the joint positions q=π and q=−π, respectively.

**Remark** **1.** *Other values for initial contractions can be adapted considering the required workspace for the robot*.

Therefore, the axial stretch of the muscles can be represented as

(24) $$\begin{array}{l} {\lambda_{z}}_{R}=\frac{l_{R}}{L_{R}}=\frac{L_{R}-\Delta L_{R_{0}}-\Delta L_{R}}{L_{R}}\\ {\lambda_{z}}_{L}=\frac{l_{L}}{L_{L}}=\frac{L_{L}-\Delta L_{L_{0}}-\Delta L_{L}}{L_{L}} \end{array}$$

where $\Delta L_{R}$ and $\Delta L_{L}$ denote the displacements of the muscles. Considering the positive direction for the joint angle as depicted in Figure 4, the displacements can be expressed in terms of q as

(25) $$\begin{array}{l} \Delta L_{R}=qr_{s}\\ \Delta L_{L}=-qr_{s} \end{array}$$

The produced torque τ as a result of the difference between the muscle forces can be formulated as

(26) $$\tau =(F_{a_{R}}-F_{a_{L}})r_{s}$$

where $F_{a_{R}}$ and $F_{a_{L}}$ are the actuation forces generated by the right and left muscles, respectively, and $r_{s}$ denotes the radius of the spool as shown in Figure 4. On the other hand, the relation (23) can be rewritten as

(27) $$F_{a}=E_{p}-\Gamma P_{air}$$

where

(28) $$\Gamma =\pi {r_{o}}^{2}-\frac{L^{2}{\lambda_{z}}^{2}}{2\pi n^{2}}$$

(29) $$\begin{array}{l} E_{P}=-\frac{2\ln(\frac{r_{o}}{r_{i}})\Phi G\pi }{\lambda_{z}}-\ln(\Omega )(-2\pi \Phi G\lambda_{z}+\frac{\Phi G\pi }{\lambda_{z}})-\\ \frac{2G{r_{o}}^{2}\pi \ln(\frac{r_{o}}{r_{i}})}{\lambda_{z}}-\frac{\left(-{r_{i}}^{2}+{r_{o}}^{2})(-4\pi a^{2}{\lambda_{z}}^{3}G-2\Phi \pi G\lambda_{z}+4\pi {r_{i}}^{2}G\right)}{2{r_{i}}^{2}{\lambda_{z}}^{2}}-\frac{0.5}{\xi } \end{array}$$

Therefore, using (26) and (27), the actuation torque is represented as

(30) $$\tau =\left(\left(E_{p_{R}}-\Gamma_{R}P_{R})-(E_{p_{L}}-\Gamma_{L}P_{L}\right)\right)r_{s}$$

If the internal pressures of the PAMs are regulated as

(31) $$\begin{array}{l} P_{R}=P_{0R}+\Delta P\\ P_{L}=P_{0L}-\Delta P \end{array}$$

where $P_{0R}$ and $P_{0L}$ are initial pressures and ΔP is the required pressure change determined by the controller, the produced torque can be written as

(32) τ=h(q)+ν(q)ΔP

where

(33) $$\begin{array}{l} h(q)=(E_{P_{R}}-E_{P_{L}})r_{s}-(\Gamma_{R}{P_{0}}_{R}-\Gamma_{L}P_{0L})r_{s}\\ \nu (q)=-(\Gamma_{R}+\Gamma_{L})r_{s} \end{array}$$

**Remark** **2.** *The initial pressures and the parameters of the controller should be set such that the desired stability region and proper performance are achieved. In the simulations, it can be seen that for some values of initial pressures, either the internal pressures or the actuation forces of the muscles, are negative for some durations. Therefore, the stability of the system is not guaranteed as the system dynamics is different from the used dynamic model in those periods. This relates to the stiffness of the joints which is highly affected by the levels of initial pressures*.

### 3.2. Equations of Motion

Equations of motion of a general n-link robot manipulator in the joints space, with disturbances written as:

(34) $$M_{r}(q)\ddot{q}+C_{r}(q,\dot{q})\dot{q}+G_{r}(q)=\tau +\tau_{d}$$

where $q,\dot{q},\ddot{q}\in R^{n}$ are the positions, velocities, and accelerations of joints, respectively, $M_{r}(q)\in R^{n\times n}$ is the inertia matrix, and $C_{r}(q,\dot{q})\in R^{n\times n}$ is the matrix of Coriolis and centrifugal forces. Also, $G_{r}(q)\in R^{n}$ denotes the vector of gravitational forces, and $\tau \in R^{n}$ and $\tau_{d}\in R^{n}$ are the input and external disturbance torque vectors of the joints, respectively. According to (31), for a PAM-actuated robot, the applied torque on the i-th joint by the associated muscles is

(35) $$\tau_{i}=h_{i}(q)+\nu_{i}(q)\Delta P_{i}$$

Consequently, the input torque vector τ can be represented in matrix form as follows

(36) τ=H(q)+V(q)ΔP

where

(37) $$\left\{\begin{array}{l} H(q)={\left[h_{1}(q_{1}),h_{2}(q_{2}),\ldots ,h_{n}(q_{n})\right]}^{T}\\ V(q)=diag[\nu_{1}(q_{1}),\nu_{2}(q_{2}),\ldots ,\nu_{n}(q_{n})]\\ \Delta P={\left[\Delta P_{1},\Delta P_{2},\ldots ,\Delta P_{n}\right]}^{T} \end{array}\right.$$

Therefore, using (34) and (35), the equations of motion for the PAM-actuated multi-link manipulator are derived in the following form:

(38) $$M_{r}(q)\ddot{q}+C_{r}(q,\dot{q})\dot{q}+G_{r}(q)=H(q)+V(q)\Delta P+\tau_{d}$$

## 4. Controller Design

In this section, an adaptive fixed time fast terminal sliding mode controller (AFFTSMC) is developed for trajectory tracking of the joints of the multi-rink PAM-actuated robots. First, a sliding surface is introduced on which the tracking error can converge to zero in a fixed time regardless of the initial conditions. Therefore, an adaptive reaching law is developed to deal with uncertainties of the model and external disturbances.

### 4.1. Fixed-Time Fast Terminal Sliding Mode

If the desired joint trajectory of the manipulator is denoted by $q_{d}$, and the trajectory tracking error is defined as $e=q-q_{d}$, the fixed-time fast terminal sliding mode s for the considered trajectory tracking problem can be formulated as

(39) $$s=\dot{e}+(c_{1}{\left|e\right|}^{k_{1}}+c_{2}{\left|e\right|}^{k_{2}})sign(e)$$

where $c_{1}$ and $c_{2}$ are positive constants, $k_{1}>1$, and $0<k_{2}<1$. The parameters can be selected by the designer to achieve the desired convergence speed and the error behaviour on the sliding surface. It can be shown that the convergence time is a Gaussian Hypergeometric Function of the surface parameters and the initial condition. It is also well known that if $k_{1}\approx k_{2}$, the function will be bounded above. In other words, if the error dynamics slide on the sliding surface (*s* = 0), it converges to the origin in a time less than or equal to a fixed time regardless of the initial conditions. That is why this sliding surface is called fixed time. Note that such control may not be practically realisable due to the fact that the control magnitude would have to be enormous to ensure that the system state from any initial condition is brought back to equilibrium within an initial-condition-independent timeframe, though mathematically, it looks elegant. For the manipulator system, to achieve fixed-time convergence, a hierarchical structure of the sliding surfaces could be used to determine the required control law for the considered system. However, this is not practically attainable even if the control signal is not limited due to uncertainties and external disturbances. On the other hand, finite-time (not fixed time) convergence can be obtained by the regular sliding mode control only if the upper bounds of the exerting torques on the joints due to the model uncertainties and external disturbances are known. In the present study, a sliding mode reaching law with an adaptive gain is developed to address this problem. Although the fixed-time convergence is not ideally achieved, the improvement of the system response is considerable, as shown in the simulations.

### 4.2. Uncertainty of the Model

It is almost impossible to determine the precise value of the inertia matrix, the matrix of Coriolis and centrifugal forces, the vector of gravitational forces, and particularly the matrix H(q). Also, the parameters gradually change over time due to either fatigue or creep. The actual values of the mentioned parameters are modeled as

(40) $$\begin{array}{l} M_{r}(q)=M_{r_{n}}(q)+\Delta M_{r}(q)\\ C_{r}(q)=C_{r_{n}}(q)+\Delta C_{r}(q)\\ G_{r}(q)=G_{r_{n}}(q)+\Delta G_{r}(q)\\ H(q)=H_{n}(q)+\Delta H(q) \end{array}$$

where the n-subscript indicates the nominal values, and the symbol Δ refers to the system uncertainties. Therefore, the system dynamics Equation (37) is rewritten as

(41) $$M_{r_{n}}(q)\ddot{q}+C_{r_{n}}(q,\dot{q})\dot{q}+G_{r_{n}}(q)=H_{n}(q)+V(q)\Delta P+\tau_{d}+F_{d}(q,\dot{q},\ddot{q})$$

where $F_{d}(q,\dot{q},\ddot{q})=-\Delta M_{r}(q)\ddot{q}-\Delta C_{r}(q,\dot{q})\dot{q}-\Delta G_{r}(q)+\Delta H(q)\in R^{n}$ is the vector of disturbance torques due to uncertainties of the system model. The recent equation can be rewritten as

(42) $$\ddot{q}=F+B_{u}u+w_{d}$$

where u=ΔP as the control input signal and

(43) $$\left\{\begin{array}{l} F={M_{r_{n}}}^{-1}(q)\left[-C_{r_{n}}(q,\dot{q})\dot{q}-G_{r_{n}}(q)+H_{n}(q)\right]\\ B_{u}={M_{r_{n}}}^{-1}(q)V(q)\\ w_{d}={M_{r_{n}}}^{-1}(q)\left[\tau_{d}+F_{d}(q,\dot{q},\ddot{q})\right] \end{array}\right.$$

### 4.3. The Control Law

In this section, the control law is proposed and it is analytically demonstrated that the controller can effectively solve the considered control problem.

**Proposition** **1.** *For the considered system and sliding surface, if* k *and* ρ *are positive constants and* $w_{d}$ *in (41) is bounded, using the control law defined as*

(44) $$u=u_{eq}+u_{s}$$

*where*

(45) $$u_{eq}={B_{u}}^{-1}\left[-F+{\ddot{q}}_{d}-(\;c_{1}k_{1}{\left|e\right|}^{k_{1}-1}+c_{2}k_{2}{\left|e\right|}^{k_{2}-1})\dot{e}-ks\right]$$

(46) $$u_{s}=-B_{u}^{-1}\gamma sign(s)$$

*with the adaption law*

(47) $$\dot{\gamma }=\rho \left|s\right|$$

*the tracking error dynamics asymptotically converges to the sliding surface, and thereby, the tracking error tends to zero eventually.*

**Proof.**  Let $\gamma_{d}$ denote the bound of $w_{d}$ and $e_{d}=\gamma -\gamma_{d}$. Then, consider the following Lyapunov function:

(48) $$V=\frac{1}{2}s^{T}s+\frac{1}{2\rho }e_{d}^{T}e_{d}$$

The time derivative of the Lyapunov function is as follows:

(49) $$\dot{V}=s^{T}\dot{s}+\frac{1}{\rho }e_{d}^{T}\dot{\gamma }$$

Using (38) and (46) yields

(50) $$\dot{V}=s^{T}\left[\ddot{e}+(c_{1}k_{1}{\left|e\right|}^{k_{1}-1}+c_{2}k_{2}{\left|e\right|}^{k_{2}-1})\dot{e}\right]+e_{d}^{T}\left|s\right|$$

which can be rewritten as

(51) $$\dot{V}=s^{T}\left[\ddot{q}-{\ddot{q}}_{d}+(c_{1}k_{1}{\left|e\right|}^{k_{1}-1}+c_{2}k_{2}{\left|e\right|}^{k_{2}-1})\dot{e}\right]+e_{d}^{T}\left|s\right|$$

Substituting (41) into (51) gives

(52) $$\dot{V}=s^{T}\left[F+B_{u}u+w_{d}-{\ddot{q}}_{d}+(c_{1}k_{1}{\left|e\right|}^{k_{1}-1}+c_{2}k_{2}{\left|e\right|}^{k_{2}-1})\dot{e}\right]+e_{d}^{T}\left|s\right|$$

Replacing u with the proposed control law leads to

(53) $$\dot{V}=s^{T}\left[-ks-\gamma sign(s)+w_{d}\right]+e_{d}^{T}\left|s\right|$$

Since $w_{d}$ is bounded by $\gamma_{d}$, the following inequality holds:

(54) $$\dot{V}\leq \gamma_{d}^{T}\left|s\right|-ks^{T}s-\gamma^{T}\left|s\right|+e_{d}^{T}\left|s\right|$$

Using the definition of $e_{d}$, (54) can be simplified to

(55) $$\dot{V}\leq -ks^{T}s$$

Therefore, from La Salle’s principle, s→0 as t→∞, which means that tracking error dynamics asymptotically reach the sliding surface and consequently, the tracking error converges to zero. □

## 5. Results and Discussion

**Figure 5:**
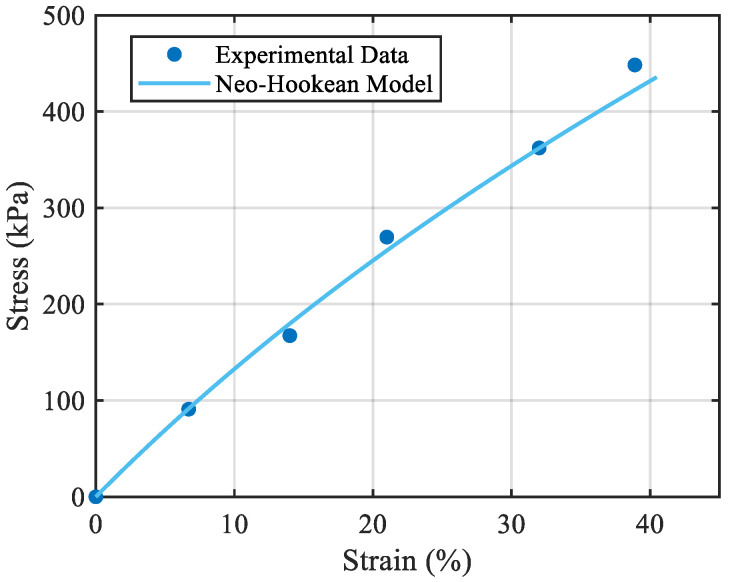
The Neo-Hookean model in comparison with the uniaxial tensile test data.

In this section, the developed continuum mechanics-based fundamental model for pneumatic artificial muscles is validated through experimental tests. The experimental work includes two different testing methods: first, testing the bladder material to determine the parameters that will be required in the mechanical model. This is carried out using a uniaxial tensile test to identify the shear modulus of the rubber bladder which is made of unfilled silicone rubber. Prior to testing, the specimens were subjected to five loading–unloading cycles to make sure that the Mullins softening was resolved [37,38]. After removing the Mullins effect, the uniaxial loading/unloading tests did not show notable hysteresis for the material in the studied loading rates, which implies that we can neglect the material’s viscoelasticity in our mechanical modelling. The results of the Neo-Hookean model are shown in Figure 5 in comparison with the test data.

The second mechanical testing method is to examine the fabricated PAM (see Figure 1) under a range of internal air pressures and axial loads. The dimensions of the actuator and the bladder material properties are reported in Table 1.

Then, the results of the numerical simulations of the performance of the proposed controller in tracking desired joint trajectories of a two-link PAM-actuated robotic manipulator are presented. The simulations were performed in MATLAB/Simulink.

**Figure 6:**
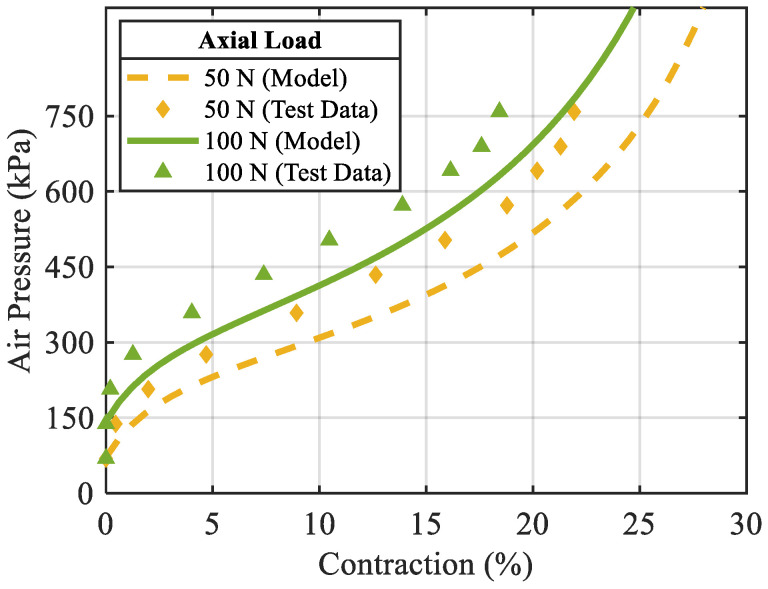
Pressure–contraction curve, comparison of the test data with the model results.

To examine the mechanical behaviour of the designed PAM, contraction of the muscle was measured against the applied air pressure while the axial force was fixed. These measurements were conducted under two axial forces applied to the muscle. The derived equations were used to predict the mechanical behaviour of the PAM, too. Figure 6 shows the comparison of the model predictions with the test data.

**Figure 7:**
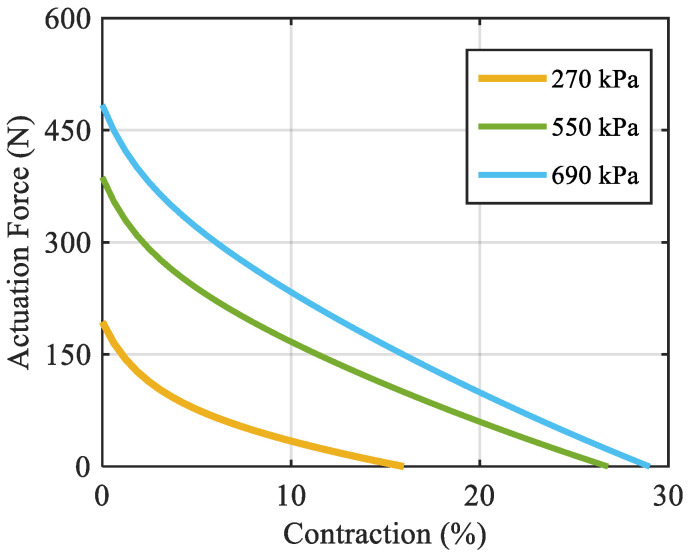
Actuation force–contraction curve at different air pressures.

The model predictions for the muscle’s actuation force are shown in Figure 7. The results show that higher air pressure leads to higher actuation force in a given contraction ratio. On the other hand, for a given load on the muscle (i.e., a certain actuation force), higher air pressure leads to higher contraction (i.e., movement of the load).

**Figure 8:**
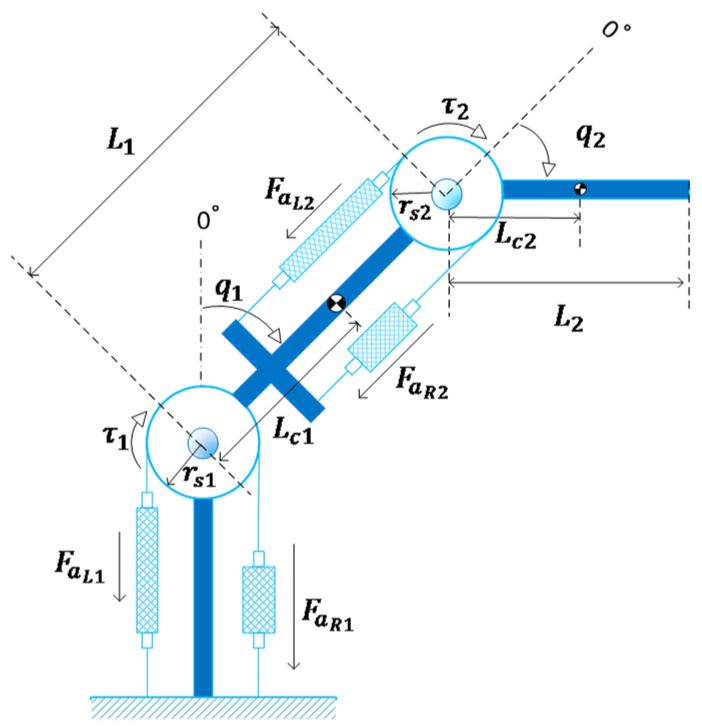
A two-link robot manipulator actuated by PAMs.

In the next step, the performance of the proposed control law for trajectory tracking control of a two-link manipulator is evaluated via numerical simulations. Figure 8 demonstrates the considered manipulator schematically.

The nominal model matrices of the manipulator are as follows:

(56) $$M_{r_{n}}=\left[\begin{array}{cc} m_{11}(q_{2}) & m_{12}(q_{2})\\ m_{21}(q_{2}) & m_{22} \end{array}\right],C_{r_{n}}=\left[\begin{array}{cc} -2c_{R}(q_{2}){\dot{q}}_{2} & -c_{R}(q_{2}){\dot{q}}_{2}\\ c_{R}(q_{2}){\dot{q}}_{1} & 0 \end{array}\right],G_{r_{n}}=g\left[\begin{array}{c} \xi_{1}(q_{1},q_{2})\\ \xi_{1}(q_{1},q_{2}) \end{array}\right]$$

where

(57) $$\begin{array}{l} m_{11}(q_{2})=m_{1}L_{c1}^{2}+m_{2}(L_{1}^{2}+L_{c2}^{2}+2L_{1}L_{c2}\cos(q_{2}))+J_{1}+J_{2}\\ m_{12}(q_{2})=m_{2}(L_{c2}^{2}+L_{1}L_{c2}\cos(q_{2}))+J_{2}\\ m_{21}(q_{2})=m_{12}(q_{2})\\ m_{22}=m_{2}L_{c2}^{2}+J_{2}\\ c_{R}(q_{2})=m_{2}L_{1}L_{c2}\sin(q_{2})\\ \xi_{1}(q_{1},q_{2})=(m_{1}L_{c1}+m_{2}L_{1})\sin(q_{1})+m_{2}L_{c2}\sin(q_{1}+q_{2})\\ \xi_{2}(q_{1},q_{2})=m_{2}L_{c2}\sin(q_{1}+q_{2}) \end{array}$$

$L_{i},m_{i}$ and $J_{i}=\frac{1}{2}m_{i}L_{i}^{2}$ are the length, mass, and approximated inertia of each link. As shown in Figure 8, $L_{ci}$ denotes the distance between the center of gravity of the *i*-th link and the *i*-th joint and is approximated by $L_{ci}=\frac{1}{2}L_{i}$.

The numerical values of the model parameters for the manipulator are presented in Table 2. The initial pressures of muscles are set to 350 kPa. The controller parameters, tuned by trial and error, are $c_{1}=diag(20,20)$, $c_{2}=diag(1,1)$, $k_{1}=1.4$, $k_{2}=0.6$, k=diag(6,6), and ρ=30.

**Remark** **3.** *The control parameters were chosen by trial and error to obtain an acceptable response. However, the following relation can be used to achieve the desired upper bound of the convergence time* $T_{\max}$ *on the sliding surface while tuning the parameters $c_{1}$, $c_{2}$, $k_{1}$, and $k_{2}$ to shape the profile of the convergence time* $T_{c}$ *over the considered region [39]:*

(58) $$T_{c}\leq T_{\max}=\frac{1}{c_{1}(k_{1}-1)}+\frac{1}{c_{2}(1-k_{2})}$$

However, even though the reaching time is finite, it is not uniform concerning the initial condition of S and the perturbation size. Moreover, it is impossible to estimate the reaching time even if there is an expression for that since it depends not only on the initial conditions but on the priori unknown upper bound of the perturbation [40]. Therefore, gains can be determined by optimising a proper cost function subject to the required constraints such as the restrictions on the maximum value of the tension in the cables, the maximum and minimum internal pressure of the muscles, etc. It is worth noting that heuristic methods such as PSO could be more efficient due to the nonlinearity of the proposed control system. Many papers have used this method to tune the sliding mode controllers of the manipulators, such as the following study [41].

The manipulator is initially at rest with the joint angles at $q_{0}={\left[0.2,0.2\right]}^{T}$. The desired trajectory of the joints is

(59) $$q_{d}={\left[\sin\left(t\right),\;0.25\sin\left(2t\right)\right]}^{T}$$

The external disturbances are assumed to be harmonic functions of time as follows:

(60) $$\tau_{d}={\left[0.08\cos(2t),\;0.04\sin(t)\right]}^{T}$$

A 10% difference between the links’ actual masses and nominal masses is assumed to evaluate the control system’s robustness against the model uncertainties.

The proposed control method compared with the developed adaptive backstepping fast terminal sliding mode controller (ABFTSMC) in [30]. To compare the performance of the controllers, similar parameters of the controllers are set equally. Furthermore, to avoid chattering, sign(s) is replaced by $\tanh(\frac{s}{\phi_{c}})$ in both controllers where $\phi_{c}$ is the approximation boundary layer and is set to 0.01.

**Figure 9:**
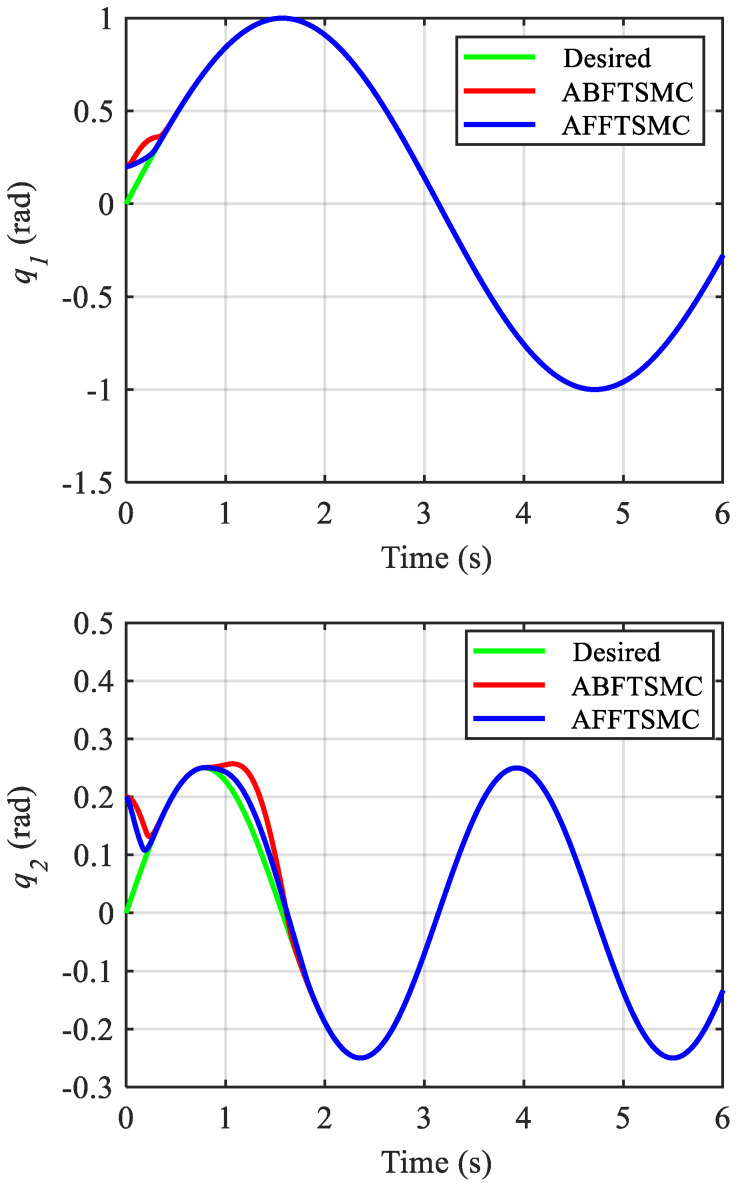
Joint angles of the manipulator.

**Figure 10:**
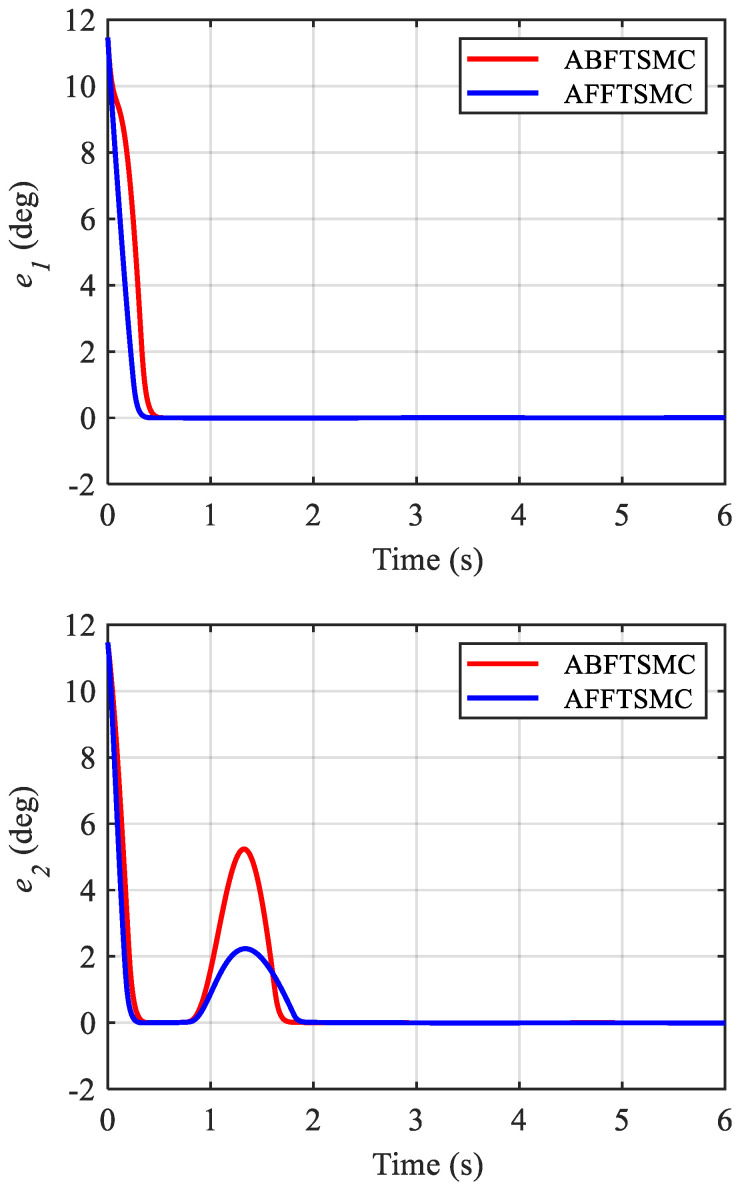
Tracking errors of the joints.

**Figure 11:**
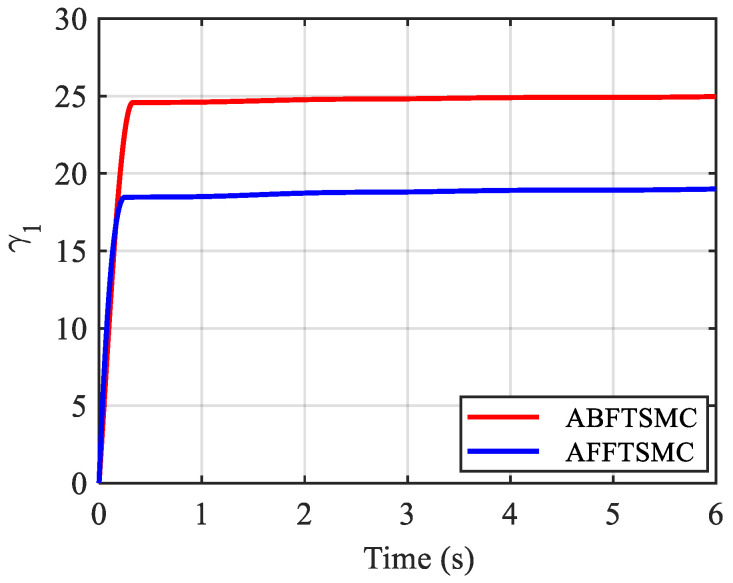

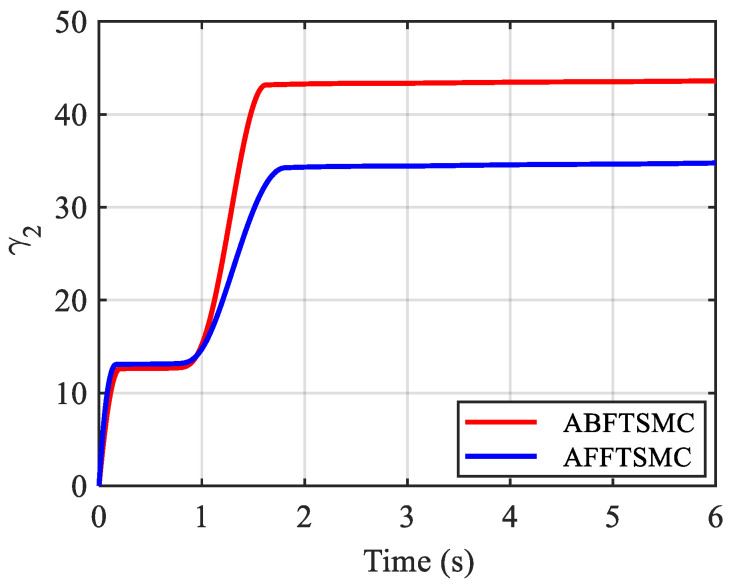
Time responses of the adaptive gains.

**Figure 12:**
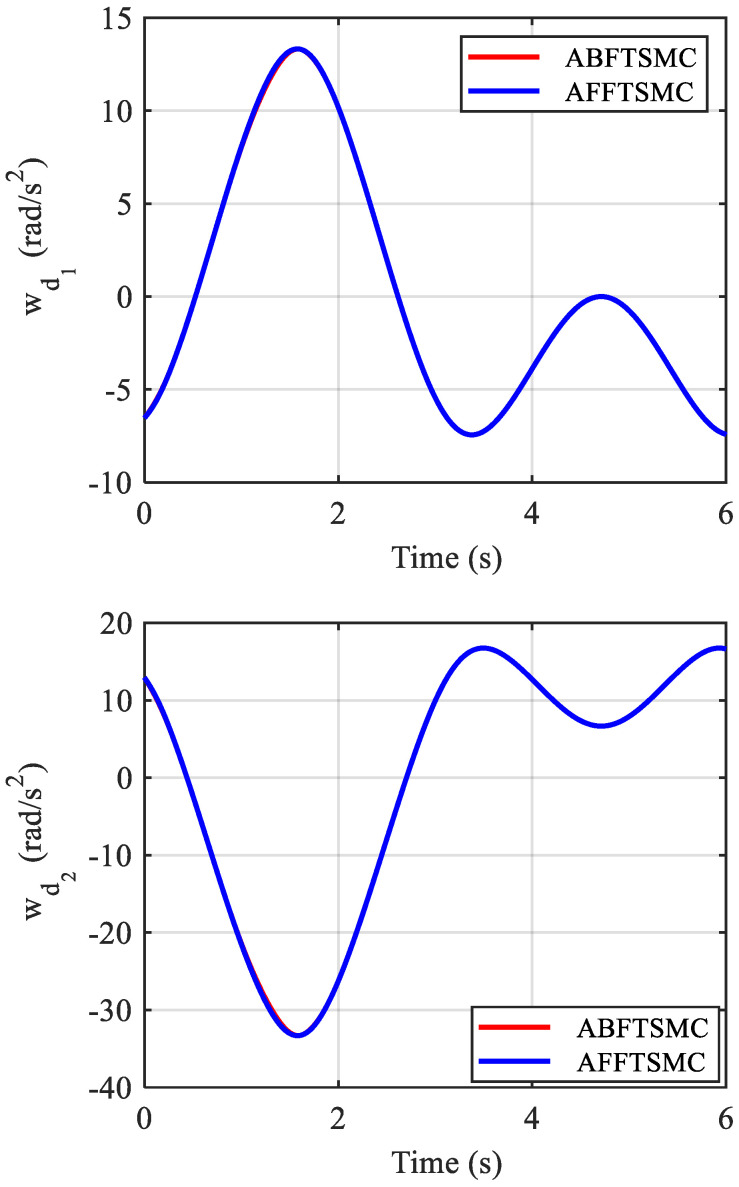
Time response of $w_{d}$.

**Figure 13:**
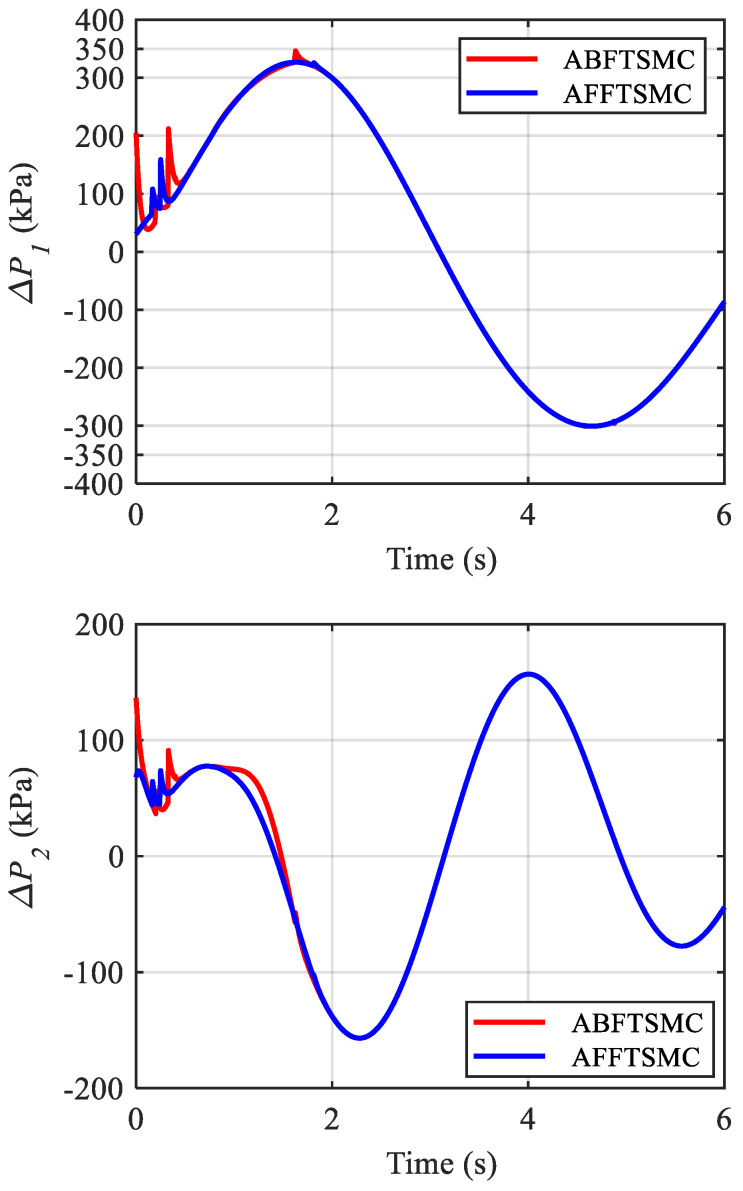
Pressure variations in the muscles.

**Figure 14:**
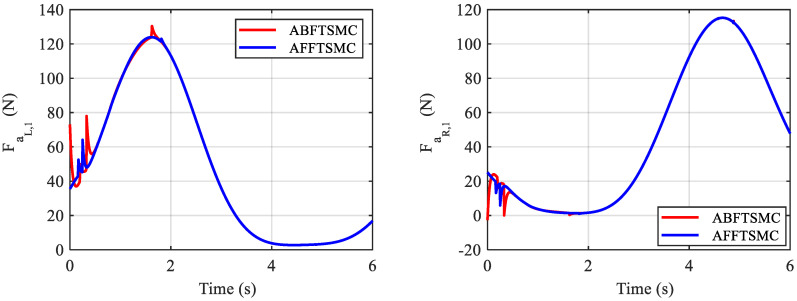

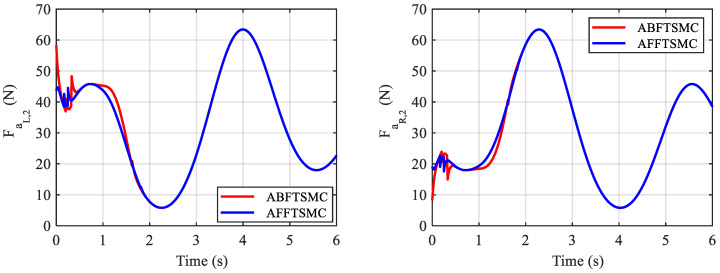
Actuation forces of the muscles.

The simulation results are shown in Figure 9, Figure 10, Figure 11, Figure 12, Figure 13 and Figure 14. Figure 9 and Figure 10 demonstrate the angle positions and the tracking errors of the joints for the proposed AFFTSMC and ABFTSMC methods. Implementing the proposed control law, the joints track their desired trajectories despite the external disturbances and the model uncertainties. Moreover, according to Figure 10, the tracking performance with the proposed controller is better than that with ABFTSMC in terms of settling time and response speed as expected. As can be seen in Table 3, the integral of the squared error (ISE) for the first joint is 10.91 deg^2^·s with the proposed controller while it is 21.52 deg^2^·s for the ABFTSMC. Moreover, the ISE for the second joint is 11.29 deg^2^·s with the AFFTSMC which is smaller than 22.59 deg^2^·s with the ABFTSMC. Therefore, tracking is improved with the proposed controller by about 50% for both joints. On the other hand, the energies of the first and second control signals with both controllers are 3.02 × 10^5^ and 5.23 × 10^4^ Pa·s, respectively. This further emphasises the effectiveness of the proposed control law as more accurate tracking is achieved with the same control energy. For both controllers, a small bump in the tracking error $e_{2}$ happens after about t=0.8 s because the adaptive gain $\gamma_{2}$, which is the estimation of the upper bound of the magnitude of $w_{d_{2}}$, converges to a value higher than its actual value until that instant, as shown in Figure 11 and Figure 12 respectively. After that instant, as the magnitude of $w_{d_{2}}$ becomes larger than $\gamma_{2}$, the tracking error increases until the adaptive gain converges to a value higher than the maximum magnitude of $w_{d_{2}}$. Then, the tracking error converges to zero once again. With the same reasoning and considering Figure 11 and Figure 12, such a bump should not be seen in $e_{1}$ for any of the controllers. Figure 10 confirms the correctness of this reasoning. As mentioned, Figure 11 demonstrates the time responses of the adaptive gains for both controllers. As can be seen, the switching gains for AFFTSMC converge to lower values. Therefore, the AFFTSMC method can use narrower boundary layers without causing the system to chatter, resulting in better tracking performance and accuracy. Figure 13 shows the pressure variation of the muscles generated by each controller. The pressure profiles for the AFFTSMC are smoother than those for the ABFTSMC, which means that the AFFTSMC is more viable in practice. Moreover, the maximum pressure variation for the AFFTSMC is less than that for the ABFTSMC. Therefore, the region of attraction for AFFTSMC may be larger than that for ABFTSMC in real-world applications where the actuator saturation limits the performance of the controllers [42]. Also, the total internal pressures of the muscles are always positive for both controllers, recalling that the initial pressure is 350 kPa. This is consistent with the assumptions of the mechanical model of PAMs. Figure 14 depicts the actuation forces of the muscles during tracking. The force values are positive with both controllers.

**Figure 15:**
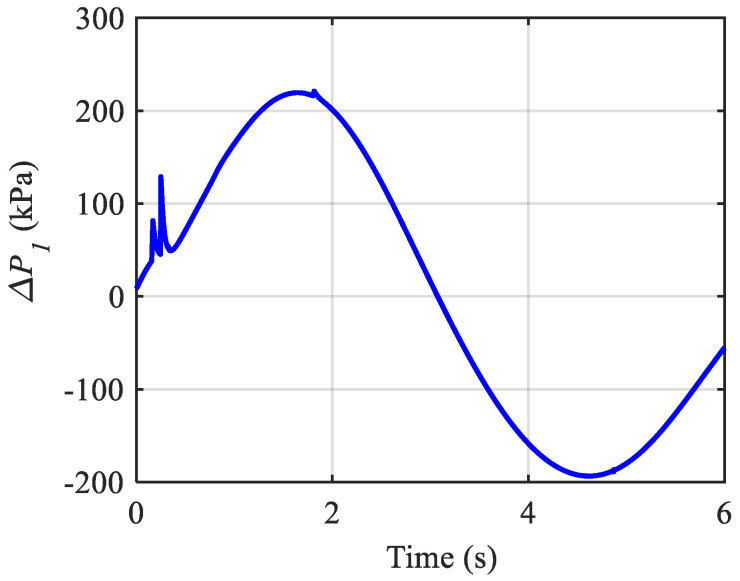
Pressure variation in the muscles of the first joint with $P_{0}=200\;\mathrm{kPa}$.

In the following, the effect of the initial pressure values on the performance and stability of the control system is evaluated. With this aim, two simulations were performed with previous gains but with the initial pressures of 200 kPa and 400 kPa. Although the angle responses and adaptive gains behaviour for the simulation with an initial 200 kPa pressure are not different and the actuation forces are all positive during the mission, the controller cannot hold the total internal pressure of the left muscle of the first joint positive for about 1 s as shown in Figure 15. This means that the control system may not be able to stabilise the trajectory of the joints on the desired trajectory. Also, even if stability is achieved, performance would not be satisfactory.

**Figure 16:**
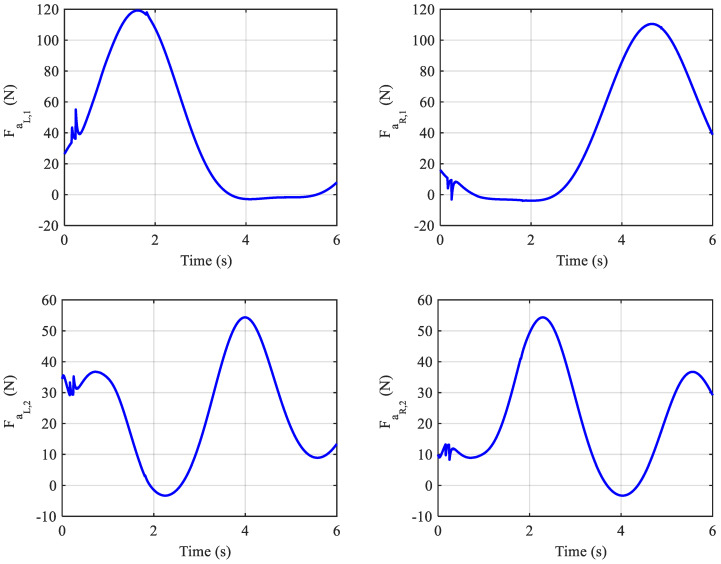
Actuation forces of the muscles with $P_{0}=400\;\mathrm{kPa}$.

With the initial pressure set to 400 kPa, the response of the angles and adaptive gains are unchanged, and the internal pressures of the muscles are held positive by the controller. But the actuation forces of the muscles need to be negative for some instances to maintain stability, as shown in Figure 16. Therefore, the system may not be stable with this internal pressure.

Therefore, the values of initial have a very important effect on the stability of the system as well as the controller parameters. In other words, the stiffness of the joints determined by these parameters plays an important role in this regard.

## 6. Summary and Conclusions

In the present work, a continuum mechanics-based model is developed for PAMs. The model provides an explicit relation between contraction ratio, air pressure, and actuation force as a set of input/output parameters for the dynamic modelling of and/or control purposes for PAM-actuated systems. Standard tensile testing of the bladder material, as well as testing a fabricated PAM under a range of internal air pressures and axial loads, was used to calibrate the proposed mechanical model. A comparison of the model predictions with experimental data shows that the model effectively predicts the mechanical behaviour of PAMs. Moreover, a dynamic model is derived for a multi-link PAM-actuated robot manipulator. To control the manipulator, which employs two antagonistic PAMs, an adaptive fixed-time fast terminal sliding mode controller is proposed to track the desired joint position trajectories. The controller is designed to cope with the model uncertainties as well as external disturbances with unknown magnitude bounds. The performance of the controller is compared with that of an adaptive backstepping fast terminal sliding mode controller. The simulations demonstrate more rapid convergence, more precise tracking, and smoother control signals with lower amplitudes for the proposed controller. In the present work, tracking only low-frequency trajectories (where the hysteresis effect is negligible) has been considered. Moreover, the friction between the bladder and threads, which is a hard nonlinearity and can affect the stability and performance of the system, has been included with other uncertainties and external disturbances in a lumped disturbance. Therefore, to enhance control system performance and to extend the application of the PAM-driven manipulators, the authors plan to further extend the mechanical model and the control method by considering the effect of material hysteresis and friction between the bladder and threads. Moreover, to extend the application of PAMs in medical and rehabilitation robots, the problem of end-effector position and stiffness control will be considered in future studies.

## Figures and Tables

**Table 1:** PAM material properties and dimensions.

μ	$R_{i}$	$R_{o}$	$\alpha_{0}$	L
217.3 kPa	2.7 mm	4.7 mm	67°	185 mm

**Table 2:** The nominal robot model parameters.

$L_{1}$	$L_{2}$	$m_{1}$	$m_{2}$	$r_{s1}$	$r_{s1}$
200 g	200 g	300 g	300 g	1.5 cm	1.5 cm

**Table 3:** Comparison of the ISE and control signal energy of the joints.

Control Method	AFFTSMC	ABFTSMC
First joint ISE	10.91	21.52
Second joint ISE	11.29	22.59
First Joint Control Signal Energy	3.02 × 10^5^	3.02 × 10^5^
Second Joint Control Signal Energy	5.23 × 10^4^	5.23 × 10^4^

## Data Availability

The original contributions presented in the study are included in the article, further inquiries can be directed to the corresponding authors.
